# Evaluating the efficiency of platform trials relative to stand-alone trials: a simulation study for the evaluation of new antibacterials

**DOI:** 10.1093/jac/dkag263

**Published:** 2026-08-04

**Authors:** Melissa J Hardy, Patrick N A Harris, Xiaofang Wang, Yin Mo, David L Paterson

**Affiliations:** Faculty of Health, Medicine and Behavioural Sciences, Frazer Institute, University of Queensland, Brisbane, Australia; Faculty of Health, Medicine and Behavioural Sciences, Frazer Institute, University of Queensland, Brisbane, Australia; Central Microbiology Laboratory, Pathology Queensland, Brisbane, Australia; Clinical Epidemiology and Biostatistics Unit, Murdoch Children's Research Institute, Melbourne, Australia; ADVANCE-ID, Saw Swee Hock School of Public Health, National University of Singapore, 12 Science Drive 2, #10-01, Singapore 117549, Singapore; Infectious Diseases Translational Research Programme, Yong Loo Lin School of Medicine, National University of Singapore, Singapore; Centre for Tropical Medicine and Global Health, Nuffield Department of Medicine, University of Oxford, Oxford, UK; Mahidol-Oxford Tropical Medicine Research Unit, Faculty of Tropical Medicine, Mahidol University, Bangkok, Thailand; Division of Infectious Diseases, University Medicine Cluster, National University Hospital, Singapore; ADVANCE-ID, Saw Swee Hock School of Public Health, National University of Singapore, 12 Science Drive 2, #10-01, Singapore 117549, Singapore; Infectious Diseases Translational Research Programme, Yong Loo Lin School of Medicine, National University of Singapore, Singapore

## Abstract

**Background:**

Serious Gram-negative bacterial infections impose significant burden, with carbapenem-resistant *Acinetobacter baumannii* and carbapenem-resistant Enterobacterales designated as WHO critical priority pathogens. Antibacterial registration trials are traditionally stand-alone two-arm randomized controlled trials evaluating one agent in a site-specific infection. Platform trials have been proposed as an alternative to identify new treatments more efficiently.

**Objectives:**

To evaluate the efficiency of different trial designs for treatments in serious resistant Gram-negative infections.

**Methods:**

We conducted simulation studies comparing platform trials with parallel and staggered starts and multiple stand-alone trials for serious infections caused by critical priority Gram-negative pathogens. Simulated recruitment and randomization were used to assess the impact of trial design, control type and allocation ratio on trial duration, sample size and operating characteristics.

**Results:**

In this setting, parallel or staggered platform trials reduced the sample size by 31–279 participants and the trial duration by 7–13 months compared with stand-alone trials. The staggered platform trial with concurrent controls required 11.0%–23.4% fewer participants and up to 46.8% less control participants than stand-alone trials. Across designs and scenarios, type I error and power, were consistently close to the nominal levels for all treatment-control comparisons.

**Conclusions:**

Platform trials can improve efficiency over stand-alone trials, reducing duration and sample size while maintaining operating characteristics. The improvement varies with control type, allocation ratio and staggered entry of agents and could help accelerate antibacterial development. This has implications for funders, sponsors and patients requiring access to new therapies, while recognizing ongoing operational and regulatory challenges.

## Introduction

Antimicrobial resistance (AMR) is a global public health challenge, with an estimated 1.14 million attributable, and 4.7 million associated deaths in 2021.^1^ Serious Gram-negative bacterial infections contribute a significant burden, with carbapenem-resistant *Acinetobacter baumannii*, carbapenem-resistant and third-generation cephalosporin-resistant Enterobacterales designated as WHO critical priorities.^1,2^ Although the antibiotic pipeline has been considered ‘dry’, the need for new agents for critical priority pathogens remains with several agents in development.^3^

Randomized controlled trials (RCTs) are the gold standard for evaluating healthcare interventions.^4^ Antibacterial registration trials are traditionally two-arm RCTs evaluating a single treatment in a site-specific infection (e.g. complicated urinary tract infection).^5^ These RCTs evaluate non-inferiority of the new agent to current standard of care. If many new antibacterials are evaluated in the same infection, each is assessed in stand-alone RCTs. For example, separate RCTs for hospital-acquired bacterial pneumonia (HABP) and ventilator-associated bacterial pneumonia (VABP) were performed for the registration of ceftazidime-avibactam, ceftolozane-tazobactam, cefiderocol and imipenem-cilastatin-relebactam.^6–9^

Many new antibacterials in development are specific for certain Gram-negative pathogens or resistance mechanisms.^3^ For example, sulbactam-durlobactam was approved in the USA and China for HABP and VABP due to *A. baumannii–calcoaceticus* complex.^10^ Three additional new antibiotics are in advanced clinical development, specifically for *A. baumannii–calcoaceticus* complex infections. The increasing focus on pathogen-directed trials in serious infections, presents substantial complexity and expense. Recruitment is challenged by severe illness, complex eligibility, diagnostic requirements and the need for numerous sites to identify patients.^11^ The sulbactam-durlobactam trial recruited 181 patients from 71 trial sites (59 sites enrolled patients) in 16 countries. Large companies have increasingly exited antibiotic development, leaving small companies reliant on public–private and non-profit organizations such as BARDA, AMR Action Fund, GARDP, CARB-X and the Wellcome Trust. In 2015, it was estimated that each patient in a registrational trial for HABP/VABP cost under $100 000.^12^ It is likely that these per-patient costs may currently be closer to $200 000.

Are there alternatives to the current paradigm of conducting individual, registrational trials? A report from the US President’s Council of Advisors on Science and Technology recommended establishing platform trials for evaluating new antibacterials, with FDA guidance and a white paper similarly suggesting exploring innovative approaches.^13–15^ A platform trial may identify effective treatments more efficiently than stand-alone RCTs, potentially reducing development time, sample size, operational costs and enabling shared infrastructure.

We conducted a simulation study to evaluate the efficiency of platform trials compared with stand-alone RCTs in the context of four antibacterials for serious infections caused by a critical priority Gram-negative pathogen. We assessed trial duration, sample size, statistical operating characteristics across different trial designs, with the aim of identifying approaches that minimize development time, participant numbers and provide earlier access to new treatments.

## Methods

The simulation study was designed using the ‘ADEMP’ framework with key features summarized in Table S1 (available as Supplementary data at *JAC* Online).^16^ The simulations compared platform trial designs with several two-arm trials across prespecified scenarios, in terms of duration, sample size and statistical operating characteristics. The effects of control type and different randomization allocations with the staggered platform design were also assessed.

### Trial designs

We simulated three trial designs: stand-alone two-arm trials, parallel and staggered platform trials. The trial design characteristics for the simulations are outlined in Table 1. The simulated trials were intended to reflect phase 3 pathogen-directed antibacterial trials for serious Gram-negative infections, including HABP, VABP or bloodstream infections.

**Table 1.** Clinical trial design characteristics and assumptions for the simulation study

Trial characteristic	Assumption
Primary endpoint	28-day ACM
Number of investigative agents	4
Staggered time (from month 0)	6, 12, 18 months
Non-inferiority margin	20% (Scenario A) and 15% (Scenario B)
Probability of mortality in control arm	41%
Probability of mortality in treatment arm	36%
Type I error	2.5%
Power	80%
Maximum enrolment per treatment arm	60 (Scenario A) and 93 (Scenario B)^a^
Sites activated per month (mean)	10
Maximum number of sites	200
Participants recruited per site per year	2
Maximum concurrent stand-alone trials at a site	2
Randomization allocation	Equal (1:1, 1:1:1:1:1), unequal (2:1:1:1:1) and adaptive (√*k*:1:1:1:1)

Further features of the simulation study are outlined in the ADEMP framework table (Table S1).

^a^The staggered platform trial with a concurrent control and a 2:1:1:1:1 allocation ratio had a maximum enrolment of 45 in the treatment arm and 90 in the control arm (Scenario A) and 71 in the treatment arm and 141 in the control arm (Scenario B).

#### Stand-alone two-arm trials

**Figure 1.**
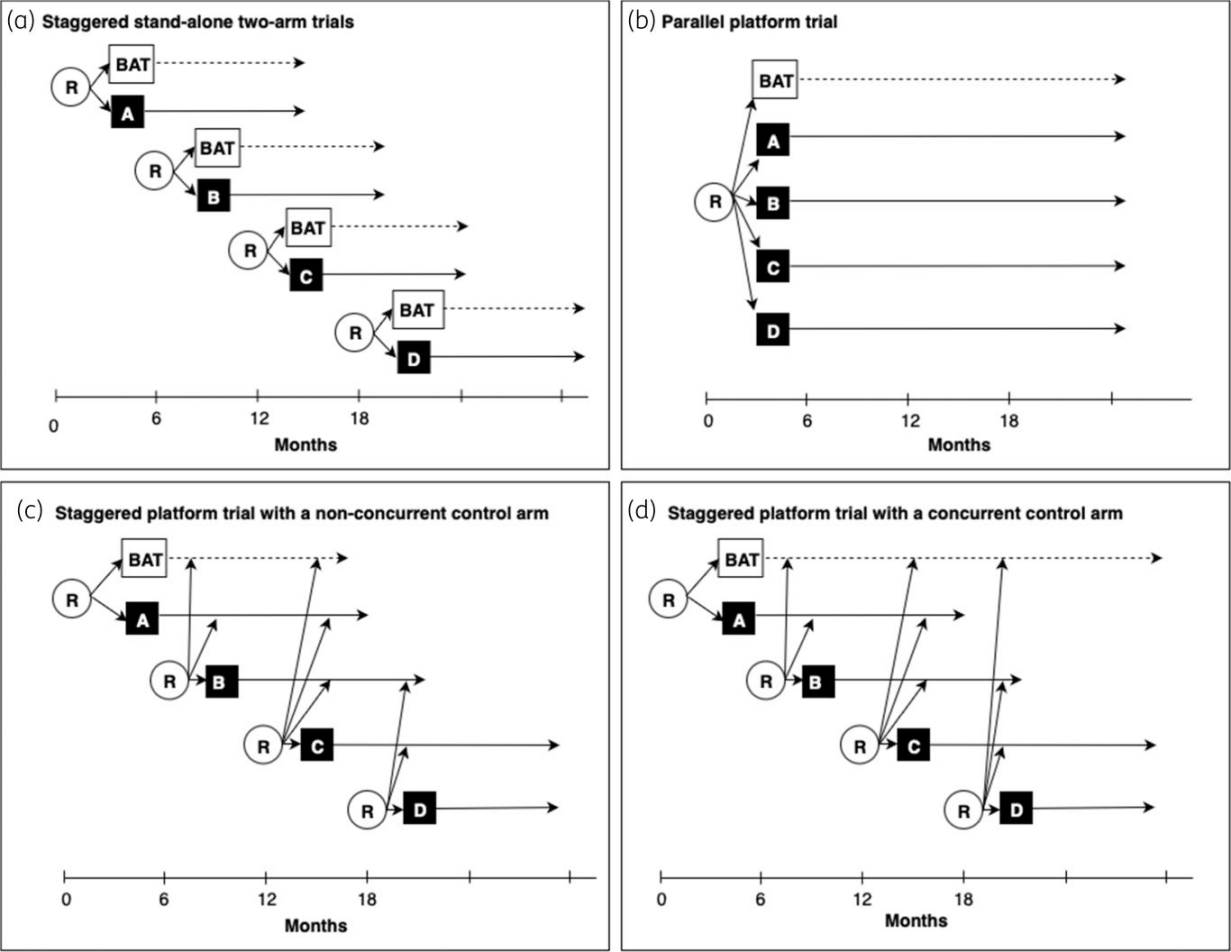
Clinical trial designs of the four staggered stand-alone two-arm RCTs (a) and the parallel (b) and staggered platform trials with a nonconcurrent (c) and concurrent control arm (d) with four investigational agents (A–D) and a control arm (BAT). Abbreviations: A–D, treatment A–D; R, randomization.

Four stand-alone two-arm trials were simulated, each comparing one drug (A–D) with best available therapy (BAT) (Figure 1a). Each trial began 6 months after the previous one, reflecting staggered initiation. For example, the trial of drug B versus BAT commenced 6 months after the trial of drug A versus BAT.

#### Platform trials

The parallel platform trial without adaptive features, evaluated all four agents against a shared control (BAT) with all arms starting concurrently (Figure 1b). By contrast, the staggered platform trial introduced a new agent into the platform every 6 months and assessed nonconcurrent (Figure 1c) and concurrent controls (Figure 1d).

### Primary endpoint and sample size

The primary outcome for this study was 28-day all-cause mortality (ACM). The effect estimate was the absolute risk difference in 28-day ACM between treatment and control groups. For all trials, non-inferiority was established if the upper bound of the two-sided 95% confidence interval for the risk difference was less than the non-inferiority margin (NIM).

Sample size calculations assumed mortality rates 41% for the control and 36% for all treatments, consistent with those used by Kaye *et al.*^10^ Two NIMs (15% and 20%) were proposed, using a type 1 error of 2.5%. The 20% margin was informed by the ATTACK trial, while the 15% margin was to evaluate a more conservative margin.^10^ This required 60 participants per group with the NIM of 20% (Scenario A) and 93 participants per group for a NIM of 15% (Scenario B), with 80% power to demonstrate non-inferiority with equal allocation within each treatment-control comparison (Table S3). No drop-out or loss to follow-up was considered.

### Randomization allocation strategies

When control comparisons are the primary focus, allocating more participants to the control arm may improve statistical power.^17,18^ We evaluated equal, unequal and adaptive allocation (i.e. 1:1:1:1:1, 2:1:1:1:1, √*k*:1:1:1:1) in the staggered platform with concurrent controls. For the adaptive allocation, participants were randomized according to a √*k*-based ratio, where *k* is the number of concurrently open treatment arms (e.g. √1 ≈1:1, √2 ≈1.41:1:1; √3 ≈1.73:1:1:1 etc.).

For the unequal allocation (2:1:1:1:1), assuming a type 1 error rate of 2.5% and 80% power, sample sizes were 45 per treatment arm and 90 control participants for Scenario A, and 71 per treatment arm and 141 control participants for Scenario B (Table S3). For the adaptive allocation of √*k*:1:1:1:1, as the allocation ratio varied dynamically based on the number of concurrent arms recruiting, the sample size requirements were the same as the equal allocation.

### Simulation study

Individual participant-level data were simulated for each trial. Each stand-alone trial was simulated independently, with staggered initiation at 6-month intervals and no sharing of recruitment, randomization or control data. Within each trial, participants were randomized 1:1 to treatment or control, ensuring balanced group sizes at the pre-determined sample sizes. The outcomes were then aggregated across all trials to provide overall estimates. Similarly, in the parallel platform trial, participants were randomized 1:1:1:1:1 across the control and four treatments, at the prespecified sample sizes.

In the staggered platform trials, a new treatment was initiated every 6 months, with participant recruitment simulated monthly and treatment assignment distributed across the control and all concurrently open treatment arms. Recruitment to each treatment arm continued until the prespecified sample size was reached. In the staggered platform trial with nonconcurrent controls, the control arm recruited the same number of participants as each treatment arm (Table S3). By contrast, the staggered design with a concurrent control, the control arm recruited throughout the trial, while each treatment arm recruited its planned sample size. This last approach helps mitigate any potential time trend bias that can arise with a nonconcurrent control.^19^

Mortality outcomes were generated using a Bernoulli distribution with the assumed mortality rates of 41% for the control and 36% with all treatments. Monthly site activation was simulated using a Poisson distribution with a mean of 10 sites per month, up to a maximum of 200 trials sites globally. Monthly recruitment for each trial was simulated with a Poisson distribution, assuming a mean of two patients per site per year. This was informed by the ATTACK trial^10^ which recruited 181 patients across 71 sites over 23 months during the COVID-19 pandemic. This corresponds to ∼1.33 patients per site-year with continuous activity. Allowing for staggered site initiation and pandemic-related disruptions, a plausible range is 1.7–2.6. In the stand-alone trials, sites could participate in up to two concurrent trials, recruiting a mean of two patients per site-year, across active recruiting sites.

Each treatment arm was compared only with the control, and no direct comparisons were made between treatment arms. In the staggered platform trial with concurrent controls, each treatment arm was compared only to the concurrent control participants. In the staggered platform trial with the nonconcurrent controls, treatment-control comparisons could include control participants enrolled before, during or after the corresponding treatment arm.

We evaluated a linear probability model (i.e. a linear regression model with a binary outcome)^20^ and logistic regression to estimate the absolute risk difference for the outcome of death (Text S1), without covariate adjustment (Text S1). The linear probability model was used because of its direct interpretability, computational efficiency and comparable simulation performance.

Each simulation was performed with 10 000 iterations as suggested by Granholm *et al.*^21^ All simulations and analyses were performed using R version 4.5.1 (R Foundation for Statistical Computing, Vienna). R codes are available at github.com/MelissaHardy/Platformtrialsimulation.

### Performance measures

We evaluated each trial design scenario in terms of efficiency and statistical operating characteristics. Efficiency measures included trial duration, total and control sample sizes, while operating characteristics were evaluated using performance metrics for treatment-control comparisons as defined in Table S2. Overall power and family-wise type I error rate (FWER) were also assessed.

## Results

### Total and control sample size

#### Stand-alone trials

**Figure 2.**
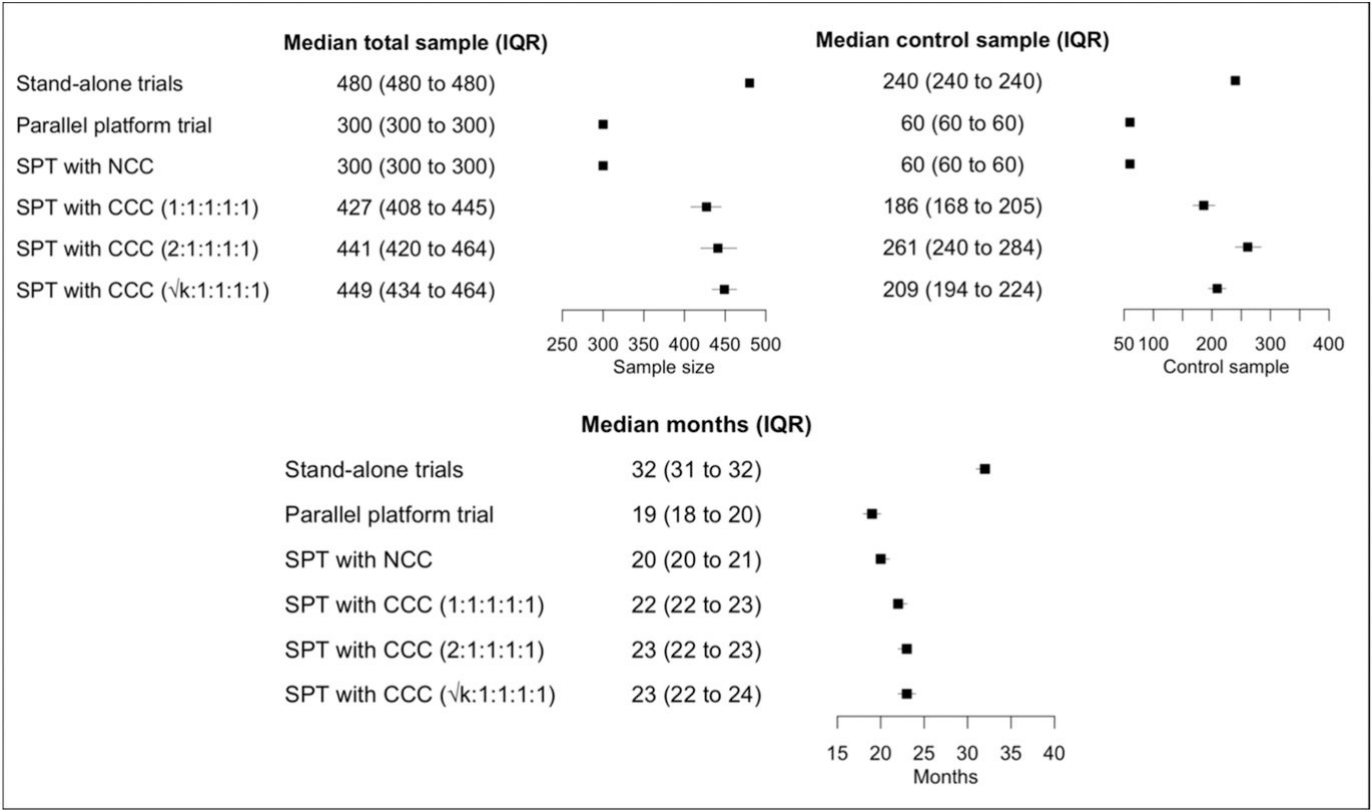
Total, control sample size and trial duration [median (IQR)] for each clinical trial design for Scenario A. Abbreviations: CCC, concurrent control; IQR, interquartile range; NCC, nonconcurrent control; SPT, staggered platform trial. Scenario A reflected a trial requiring 60 participants per arm, corresponding to a 20% NIM.

**Figure 3.**
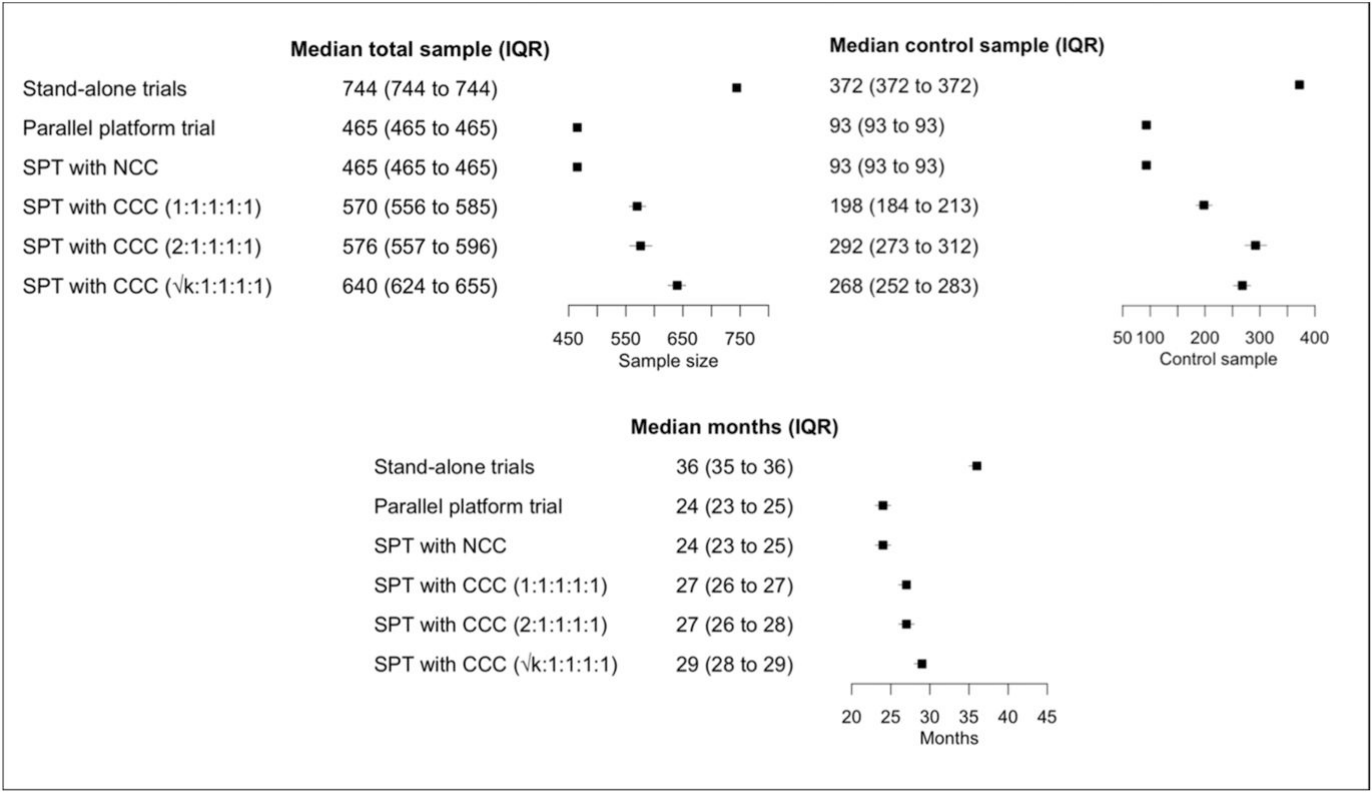
Total, control sample size and trial duration [median (IQR)] for each clinical trial design for Scenario B. Abbreviations: CCC, concurrent control; IQR, interquartile range; NCC, nonconcurrent control; SPT, staggered platform trial. Scenario B reflected a trial requiring 93 participants per arm, corresponding to a 15% NIM.

The total and control sample size for each stand-alone trial was 60 per arm with the NIM of 20% (Scenario A) and 93 for the NIM of 15% (Scenario B), as outlined previously (Table S3). The total enrolment for the four stand-alone trials was 480 (120 per trial) and 744 participants (186 per trial), respectively (Figures 2 and 3).

#### Platform trial designs

The parallel platform trial required 300 participants for a NIM of 20% (Scenario A), with 60 participants per control and each treatment arm. For a NIM of 15% (Scenario B), it required 465 participants, with 93 participants per control and each treatment arm.

The staggered platform trial with nonconcurrent controls, required the same total sample size with 300 in Scenario A and 465 in Scenario B. This corresponded to 60 and 93 participants per arm, respectively. The staggered platform trial with concurrent controls required larger total and control sample sizes than the other platform designs. With equal allocation, our simulations resulted in median total sample sizes of 427 (IQR 408–445) in Scenario A and 570 (IQR 556–585) in Scenario B; median control sample sizes were 186 (IQR 168–205) with 60 per treatment arm and 198 (IQR 184–213) with 93 per treatment arm, respectively. With an unequal allocation (2:1:1:1:1), median total sample sizes were 441 (IQR 420–464) for Scenario A and 576 (IQR 557–596) for Scenario B. The corresponding median control sample sizes were 261 (IQR 240–284) with 60 per treatment arm and 292 (IQR 273–312) with 93 per treatment arm, respectively. For the adaptive allocation (√*k*:1:1:1:1), median total sample sizes were 449 (IQR 434–464) for Scenario A and 640 (IQR 624–655) for Scenario B. This corresponded to median control sample sizes of 209 (IQR 194–224) with 60 per treatment arm and 268 (IQR 252–283) with 93 per arm, respectively (Figures 2 and 3).

Thus, overall, the four stand-alone trials required the most total and control participants. As expected, there was a total requirement of 480 (60 in each of four investigational drug arms and 240 controls) for Scenario A and 744 participants (93 in each of four investigational drugs arms and 372 controls) for Scenario B. An exception was observed for Scenario A, where the staggered platform trial with unequal allocation required more control participants than the stand-alone trials [median of 261 (IQR 240–284) versus with 240 (IQR 240–240)], whereas for Scenario B the same design required fewer control participants [median 292 (IQR 273–312)] than the stand-alone trials [median 372 (IQR 372–372)]. Overall, the staggered platform trials with concurrent controls required up to 11.0% and 23.4% fewer total participants than the stand-alone trials for Scenarios A and B (Figures 2 and 3). However, with this design, in up to 18.5% of simulations, equal or unequal allocation yielded control groups with fewer than expected participants (Table S4). Under the adaptive allocation, total sample size reductions versus the stand-alone trials was modest (6.5% and 14% in Scenarios A and B).

### Total trial duration

#### Stand-alone trials

The median duration of the first trial, comparing drug A with BAT, was 13 months (IQR 12–14) with Scenario A (20% NIM) and 17 months (IQR 16–19) with Scenario B (15% NIM). With the 6-monthly staggered trial initiation, the median trial duration for the drug B trial was 15 months (IQR 14–15) in Scenario A and 20 months (IQR 19–21) in Scenario B. The median duration for the drug C trial was 15 months (IQR 14–15) with Scenario A and 19 (IQR 18–19) months with Scenario B. The median duration the drug D trial was 14 months (IQR 13–14) with Scenario A and 18 months (IQR 17–18) with Scenario B (Figure S1). All four trials were completed in a median of 32 months (IQR 31–32) after first enrolment in the drug A trial with Scenario A and 36 (IQR 35–36) months with Scenario B (Figures 2 and 3).

#### Platform trial designs

All platform trial designs had shorter total durations than the four stand-alone RCTs, from 9–13 months less for Scenario A and 7–12 months for Scenario B (Figure 2 and 3). The parallel platform trial was shortest with a median trial duration of 19 (IQR 18–20) and 24 months (IQR 23–25) for Scenario A and B, respectively. The staggered platform trial with nonconcurrent controls was similar (20 (IQR 20–21) and 24 months (IQR 23–25)). The staggered platform trial with concurrent controls were up to 10 months shorter (22 (IQR 22–23) and 27 months (IQR 26–27)) than the four stand-alone trials. Total median duration was similar for both equal and unequal allocation (22–23 and 27 months). The adaptive allocation increased the duration by 2 months for Scenario B. The median duration was 4–6 months longer for Scenario B than for Scenario A for each trial design, reflecting the larger sample size requirement with a 15% NIM.

### Type I error and power

For each treatment-control comparison, trial design and scenario, the type I error rate was close to 0.025, ranging from 0.019 to 0.028, with small Monte Carle standard errors (MCSE) of <0.003 (Table 2). The power for each treatment-control and trial design was consistent with 0.80, from 0.795 to 0.888 for both scenarios. The staggered platform trial with adaptive allocation slightly increased the subgroup power (0.862 to 0.872 in Scenario A; 0.870 to 0.888 in Scenario B), probably due to increased allocation to the control arm. The FWER was mostly lower for the platform trials (0.01 to 0.019 in Scenario A and 0.002 to 0.022 in Scenario B), and lowest for the staggered platform trial and nonconcurrent controls in both scenarios. The overall power was reduced with the platform trials compared with stand-alone trials. The exception was the staggered platform trial with adaptive allocation, where power was similar across scenarios.

**Table 2.** Type I error rate and power for each specific treatment (Drug A–D) and control comparisons, FWER and overall power

	Scenario A					Scenario B				
Trial design	Drug A	Drug B	Drug C	Drug D	FWER	Drug A	Drug B	Drug C	Drug D	FWER
Type I error rate (MCSE < 0.002 for drug-control comparisons and <0.003 for FWER)										
Stand-alone trials	0.025	0.027	0.025	0.026	0.099	0.024	0.023	0.024	0.025	0.092
Parallel platform trial	0.028	0.025	0.026	0.024	0.080	0.021	0.021	0.022	0.026	0.070
SPT—NCC	0·027	0·025	0·026	0·027	0·08	0·026	0·025	0·024	0·026	0·078
SPT—CCC (1:1:1:1:1)	0·023	0·021	0·021	0·024	0·082	0·026	0·025	0·024	0·023	0·088
SPT—CCC (2:1:1:1:1)	0·023	0·019	0·025	0·026	0·089	0·025	0·026	0·026	0·023	0·094
SPT—CCC (√*k*:1:1:1:1)	0·020	0·026	0·024	0·022	0·086	0·024	0·025	0·025	0·024	0·087

Trial design	Drug A	Drug B	Drug C	Drug D	Overall power	Drug A	Drug B	Drug C	Drug D	Overall power
Power (MCSE < 0.004 for drug-control comparisons and <0.002 for overall power)										
Stand-alone trials	0·809	0·807	0·800	0·811	0·999	0·799	0·795	0·802	0·803	0·999
Parallel platform trial	0·810	0·810	0·815	0·813	0·976	0·797	0·795	0·795	0·799	0·972
SPT—NCC	0·804	0·812	0·806	0·809	0·974	0·800	0·800	0·796	0·798	0·970
SPT—CCC (1:1:1:1:1)	0·809	0·812	0·820	0·844	0·997	0·808	0·814	0·817	0·828	0·992
SPT—CCC (2:1:1:1:1)	0·808	0·815	0·813	0·821	0·996	0·810	0·809	0·813	0·821	0·994
SPT—CCC (√*k*:1:1:1:1)	0·864	0·872	0·872	0·862	0·999	0·870	0·888	0·888	0·876	0·998

CCC, concurrent control; NCC, nonconcurrent control; SPT, staggered platform trial added a new drug to the platform every 6 months.

### Simulation performance

The operating characteristics across all trial designs and scenarios are outlined in Table S5. The relative bias (%) was small, ranging from −4.19 and 4.42%, with MCSEs of <1.8% (Table S5). The empirical standard error for each subgroup and overall was modest, with 0.09 for Scenario A and 0.07 for Scenario B. The only exception was the staggered platform trial design with adaptive allocation, where it was slightly lower for each scenario (0.08 and 0.06 for Scenarios A and B). The percentage relative error in the model-based standard error was consistently low, from −1.61 to 0.55. The coverage probabilities were close to the nominal 95% level (94.3%–95.2%) and the mean standard error ranged from 0.006 to 0.008 for Scenario A and 0.004 to 0.005 for Scenario B.

## Discussion

Our simulations demonstrate platform trials with parallel and staggered start with four agents can be more efficient than four stand-alone RCTs. These improvements were seen in duration, total and control sample size. We also observed the impact on efficiency with the staggered platform trial, using a nonconcurrent and concurrent control, equal, unequal and adaptive allocation. Such efficiencies may help streamline antibacterial development in pathogen-directed trials compared with multiple two-arm trials, although broader operational, funding and regulatory considerations remain.

We found the parallel platform and staggered platform trials with a nonconcurrent control were the shortest and required the least total and control participants. However, given the limited pipeline of new antibacterials targeting drug-resistant pathogens, it may be infeasible for simultaneous availability of four agents for a parallel platform design.^3^ However, this design could include established antibacterials or treatment strategies as comparators to provide additional information to clinicians. With the staggered platform trial with nonconcurrent controls, evolving standards of care, shifts in disease epidemiology, advances in rapid diagnostic technologies or changes in resistance profiles may introduce bias from temporal changes as treatment arms are added.^19,22^ The FDA regulatory guidance on master protocols currently prefers the use of concurrent controls, however, there may be situations where nonconcurrent controls may be acceptable (i.e. rare diseases, paediatrics or an unmet medical need).^23^

The staggered platform designs with a concurrent control were also shorter in duration with less total and control participants. In some simulations, equal and unequal allocation yielded fewer control participants than required, potentially reducing power. The adaptive allocation increased trial duration, sample size and control participants, but was associated with higher power. FDA regulatory guidance recommends consideration of a randomization scheme that allocates more participants to the control as this can increase power.^23,24^ However, allocating more participants to the control arm may raise ethical concerns, particularly with drug-resistant infections with limited treatment options, life-threatening infections and toxicity concerns with older comparator antibiotics. While there have been recent antibacterial trials that included a 2:1 randomization with the new agent versus the control, these trials were primarily descriptive studies and were not powered for efficacy hypothesis testing.^25–27^ The smaller NIM in Scenario B required a larger sample size, which increased the trial duration and sample size across all designs. Further reducing the margin (e.g. to 5%) would substantially lengthen trial duration and increase sample size. This illustrates the compromise between narrower margins that provide greater assurance of efficacy over the feasibility of conducting timely and efficient trials.

The operating characteristics of each design and scenario demonstrated the type I error can be consistently maintained for each treatment-control subgroup. The FWER was mostly lower for the platform designs than that of the stand-alone trials. There is ongoing debate on the necessity for FWER adjustment for multiplicity in platform trials with several treatments and a shared control.^19^ Multiplicity corrections may not be required for independent treatment-control comparisons in platform trials.^23,28^ However, exceptions may apply at different doses, administration modes or combination therapies.^23^

Our simulations showed that platform trials can improve efficiency, through shared control arms and infrastructure to evaluate multiple treatments. This approach has been applied in several therapeutic areas such as COVID-19, oncology, amyotrophic lateral sclerosis, Alzheimer’s disease and proposed by the EU Patient-Centric Clinical Trial Platforms (EU-PEARL) in diseases to overcome development challenges when facing unmet medical need.^19,29–33^ However, serious bacterial infections may present additional challenges for platform trials with heterogenous infection syndromes, acute clinical deterioration, variability in pathogen identification and background therapy. Platform trials also require greater expertise, infrastructure and upfront investment to establish and maintain the platform framework.^34^ Alternative funding sources may be required, potentially from combined government, industry, academia and not-for-profit sources. Important regulatory considerations, as highlighted by the EU-PEARL consortium,^19^ remain, alongside evolving regulatory guidelines, such as the acceptance of a single pivotal trial and the FDA draft guidance on the use of Bayesian methodology in clinical trials, further influencing future antibacterial trial design and development pathways.^35,36^

Our study has several limitations. Our simulations were based on a simplified pathogen-directed trial design informed by the sulbactam-durlobactam trial,^10^ a recent example of pathogen-directed antibacterial development, and may not fully reflect the evolving regulatory landscape and all pathways for antibacterial development. Some new antibacterials may retain broad-spectrum activity or pharmacodynamic advantages where site-specific trials may be required with pathogen-directed approaches. Our findings depend on assumptions about enrolment rates and treatment effects and may not generalize to other settings. Our simulations considered four new agents, and different numbers of agents may affect efficiency. We did not incorporate interim analyses or adaptive features, which can enable early efficacy and futility decisions and further improve efficiency. Although not currently used in antibacterial registration trials, adaptive features such as response-adaptive randomization and multi-arm multi-stage designs may have further impact. We used a frequentist approach; further evaluation of Bayesian methods commonly used in platform trials may provide additional insights.

### Conclusions

Our simulation study demonstrated efficiency of parallel and staggered platform trials over stand-alone trials in the setting of antibacterials for pathogen-directed serious infections. We found that platform trials can reduce the total and control participants and duration. The advantages vary with the staggered addition of agents, control type and allocation ratios. Such efficiencies may have the potential to streamline the development antibacterials for critical priority pathogens, compared with multiple traditional two-arm trials. However, further consideration of operational feasibility, methodological challenges and regulatory considerations remain for the broader implementation of platform trial designs in this setting.

## Supplementary Material

dkag263_Supplementary_Data

## Transparency declarations

D.L.P. has research funding from Shionogi, Merck, Gilead, bioMerieux, BioVersys and Pfizer and has received consulting fees from the AMR Action Fund, CARB-X, Aurobac, Innoviva, GSK, Pfizer, Merck, Cepheid, bioMérieux, Tamrisa, BioVersys and Spero. M.J.H. is a shareholder of Pfizer and has received consulting fees from argenx. P.N.A.H. reports research support from Gilead, Microbio and Tamrisa and has received honoraria from Shionogi, bioMerieux, Echo Therapeutics and Pfizer. All other authors: none to declare.

## Supplementary data

Text S1, Figure S1 and Tables S1 to S5 are available as Supplementary data at *JAC* Online.
