# Supplementary material for: Evaluating the efficiency of platform trials relative to stand-alone trials: a simulation study for the evaluation of new antibacterials
Source: J Antimicrob Chemother. 2026 Aug 4;81(9):dkag263. doi: 10.1093/jac/dkag263 (PMC13435618; doi:10.1093/jac/dkag263)
Supplement: Supplementary file 1 [file dkag263_supplementary_data.docx]

**Supplementary Appendix – Hardy et al, Evaluating the efficiency of antibacterial platform trials over multiple stand-alone trials: A simulation study for the evaluation of new antibacterials**

**Contents:**

1. **Table S1.** Overview of the key features of the simulation study using the ADEMP framework
2. **Text S1:** Evaluation of Analysis Methods
3. **Table S2:** Summary and definition of performance measures
4. **Table S3:** Proposed sample sizes for simulated trial designs.
5. **Figure S1.** Trial duration for each stand-alone trial
6. **Table S4:** Proportion and mean of low control comparisons with the staggered platform trial with different allocation ratios.
7. **Table S5:** Simulation performance measures.

**Table S1. Overview of the key features of the simulation study using the ADEMP (aims, data-generating mechanism, estimands and other targets, methods, performance measures) framework**

| **Key features** | **Simulation study** |
| --- | --- |
| Aims | - To assess the efficiency of different platform trials designs compared with multiple stand-alone two arm randomised controlled trials in terms of trial duration, total and control sample size and trial sites. - To investigate the impact of concurrent and nonconcurrent control arms, and equal, unequal and adaptive randomisation allocation ratios with a staggered platform trial. - To evaluate the impact of each trial design scenario on the statistical operating characteristics such as Type 1 error rate and power. |
| Data generating mechanism | - Treatment was randomly assigned using either fixed permutation (for standalone and parallel platform trials) or a multinomial distribution (for staggered platform trials), according to the allocation ratio defined by each trial design scenario - Mortality outcomes of the data were drawn from a binomial distribution with the predicted treatment outcomes - The number of sites activated, and patients enrolled per month were drawn from a Poisson distribution |
| Estimand | - Risk difference with the proportion with mortality rates with the investigational agents and control - Non-inferiority will be established if the upper bound of the two-sided 95% confidence interval for the risk difference is less than the non-inferiority margin - Trial logistics: total duration, total sample size, control sample size, number of sites initiated |
| Method of analysis | Linear probability model will be applied to the data to obtain the risk difference and 95% CI of the primary outcome |
| Performance measures | - Group-specific type 1 error rate and overall type 1 error - Group-specific power and overall power - Additional measures: Relative bias, Coverage probability, Mean squared error, Empirical standard error, Relative Error in Model-Based standard error |
| Number of repetitions | 10,000 |

**Text S1: Evaluation of Analysis Methods**

For this simulation study, we compared a logistic regression model and a linear probability model to determine the most appropriate method of analysis for estimating treatment effects on a binary outcome in a randomised controlled trial (RCT) without covariate adjustment. The effect estimate was the absolute risk difference in 28-day all-cause mortality between the treatment and control arms. Logistic regression is widely used with binary outcomes and provide estimates of an odds ratio. To obtain a risk difference, the coefficients must be converted from the log-odds scale to probabilities.[1] The linear probability model is a linear regression model for a binary outcome, i.e. for a dependent variable that is either zero or one, and produces coefficients that can be directly interpreted as risk differences. [1,2] However, the linear probability model has some recognised limitations. It may produce predicted probabilities outside the 0 and 1 range, particularly in more complex settings. [2,3] In addition, heteroscedasticity may occur, which can be addressed by using robust standard errors. [2,3]

We simulated individual-level data for participants in a two-arm stand-alone RCT. Participants were randomly allocated to the treatment or control arm with equal probability and each participants outcome for the presence or absence of death was randomly generated from a Bernoulli distribution with the assumed mortality rates of 0·36 and 0·41 for the treatment and control arms. The sample sizes were based on the simulation study estimates, with 120 and 186 participants per study with a 20% and 15% non-inferiority margin.

To estimate the treatment effect, both a logistic regression model and a linear probability model were applied. The logistic regression model was specified as:

$$P\left( Y_{i} =1 \right)={logit}^{-1}(\beta_{0} + \beta_{1}D_{i} )$$

where $P\left( Y_{i} =1 \right)$ denotes the probability of the outcome for participant *i*, $D_{i}$ is the treatment assignment, $\beta_{0}$ is the baseline log-odds of the outcome in the control group, and $\beta_{1}$ represents the treatment effect on the log-odds scale.[2] The logistic regression model assumed a binomially distributed outcome with a logit link function, producing a coefficient that could be interpreted as an odds ratio. Predicted probabilities were used to calculate the risk difference between treatment and control groups, and bootstrapping was applied to derive confidence intervals for these estimates as described by Yorlets et al. [4]

The linear probability model was specified as:

$$Y_{i}= \beta_{0} + \beta_{1}D_{i}$$

where $Y_{i}$ is the binary outcome for participant *i,* $D_{i}$ is the treatment assignment, $\beta_{0}$ is the baseline risk in the control group, and $\beta_{1}$ is the estimated absolute risk difference between treatment groups. [2] The linear probability model was fitted with a Gaussian distribution with an identity link function and robust standard errors, producing a coefficient that was directly interpreted as a risk difference.

To compare the two methods, we analysed the simulated data to estimate the risk difference, standard error and 95% confidence interval across 1000 simulations. This exploratory comparison was conducted separately from the primary simulation analyses, which used 10,000 iterations. The performance of both approaches was also assessed in terms of relative bias (mean percent error), coverage probability (proportion of simulations with a confidence interval that includes the true risk difference) and precision (average width of the confidence interval). All analyses were performed in R version 4·5·1 (R Foundation for Statistical Computing, Vienna).

The simulation results for the risk difference estimates for both sample sizes are shown in Table 1. The two models achieved similar estimates of the risk difference and confidence intervals for both sample sizes, with negligible difference in the standard errors. The distribution of the risk difference in Figure 1, illustrates both methods of analysis perform comparably. In terms of performance measures (Table 2), both models for each sample size demonstrated similar results. With the smaller sample size, both methods showed minimal bias with consistent estimation. At the larger sample size, the methods diverged in their estimates, although the relative bias remained low. The coverage probability was similar and close to the 95% nominal level for both sample sizes and models. The precision of both methods was nearly identical at each sample size. Confidence intervals narrowed from approximately 0·347 at the smaller sample size to 0·279 at the larger, reflecting improved precision with the larger sample.

While both the logistic regression and linear probability model provided consistent and unbiased estimates, the logistic regression model required substantially more processing time, most likely due to the use of bootstrapping to derive confidence intervals. The generalised linear model with an identity link using robust standard errors was selected for the simulation study due to its direct interpretation as a risk difference, unbiased estimates, ease of implementation and more efficient processing time.

**Table 1**. Risk difference estimates for the logistic regression and linear probability model with the two sample sizes from 1000 simulations

| **Sample Size** | **Method** | **Risk Difference** | **SE** | **95% CI** |
| --- | --- | --- | --- | --- |
| 120 | Logistic Regression | -0·0509 | 0·0887 | (-0·224, 0·124) |
|  | LPM | -0·0507 | 0·0883 | (-0·224, 0·122) |
| 186 | Logistic Regression | -0·0471 | 0·0713 | (-0·187, 0·093) |
|  | LPM | -0·0523 | 0·0710 | (-0·191, 0·087) |

Abbreviations: CI, Confidence interval; LPM, Linear probability model; SE, Standard error;

**Table 2.** Performance measures for the logistic regression and linear probability model with the two sample sizes from 1000 simulations

| **Sample Size** | **Method** | **MPE** | **CP** | **Precision** |
| --- | --- | --- | --- | --- |
| 120 | Logistic Regression | 1·85 | 94·3 | 0·348 |
|  | LPM | 1·33 | 94·9 | 0·346 |
| 186 | Logistic Regression | -5·84 | 94·8 | 0·280 |
|  | LPM | 4·60 | 92·5 | 0·278 |

Abbreviations: CP, Coverage probability; LPM, Linear probability model; MPE, Mean percent error;

**Figure 1.** Illustration of the estimates of the risk difference for the logistic regression and linear probability model with each sample size. The grey dashed line indicates the true risk difference of -0·05. These figures display the estimates from 1,000 simulations.

**References:**

1. Aldrich J, Nelson F. Linear Probability, Logit, and Probit Models, SAGE Publications, Inc.; 1984. Available from: <https://methods.sagepub.com/book/linear-probability-logit-and-probit-models> doi:[10.4135/9781412984744](https://doi.org/10.4135/9781412984744)
2. Gomila R. Logistic or linear? Estimating causal effects of experimental treatments on binary outcomes using regression analysis. J Exp Psychol Gen **2021**; 150:700–709.
3. Naimi AI, Whitcomb BW. Estimating Risk Ratios and Risk Differences Using Regression. Am J Epidemiol **2020**; 189:508–510.
4. Yorlets RR, Lee Y, Gantenberg JR. Calculating risk and prevalence ratios and differences in R: developing intuition with a hands-on tutorial and code. Ann Epidemiol **2023**; 86:104–109.

**Table S2. Summary and definition of the performance measures**

| **Performance Measure** | **Definition** | |
| --- | --- | --- |
| Total trial duration | Median and IQR months from trial initiation to last participant randomised | |
| Total sample size | Median and IQR of participants required for the control arm and all four treatment arms | |
| Total control sample size | Median and IQR of control participants required for the trial design | |
| Total sites initiated | Median and IQR of sites initiated for the trial design before completing recruitment | |
| Power | Proportion of iterations concluding non-inferiority for a treatment-control comparison | |
| Type I error rate | Proportion of iterations incorrectly concluding non-inferiority when inferior outcomes for the treatment arm (mortality outcome of 61%) were generated for a treatment-control comparison. In each iteration, the upper bound of the 95% confidence interval was compared to the non-inferiority margin and where it was less than the margin, were counted as type I errors. | |
| Overall power | Proportion of iterations where one or more treatment arms concluded non-inferiority | |
| Family-wise type I error rate | Proportion of iterations where one or more treatment arms incorrectly concluded non-inferiority with inferior outcomes for all four treatment arms | |
| Relative bias | Difference between the mean estimated risk difference and the true risk difference, divided by the true risk difference. Indicates the mean percent of simulations that over or underestimate the true risk difference. | $\frac{(\frac{1}{n_{sim}} \sum_{i=1}^{n_{sim}} \hat{\theta_{i}})-\theta}{\theta}$ |
| Empirical SE | Measure of precision, calculated as the standard deviation of the estimated risk differences for all simulation iterations. | $\sqrt{\frac{1}{n_{sim}-1}\sum_{i=1}^{n_{sim}} {(\hat{\theta}_{i}- \bar{\theta})}^{2}}$ |
| Mean squared error | Mean of the squared difference between the estimated and true risk difference which provides a measure of model performance. | $\frac{1}{n_{sim}}\sum_{i=1}^{n_{sim}} {(\hat{\theta}_{i}- \theta)}^{2}$ |
| Relative % error in model-based standard error | Percentage difference between the model-based SE and the empirical SE, expressed relative to the empirical SE. Positive values indicate overestimation by the model; negative values indicate underestimation. | $100\left( \frac{\hat{ModSE}}{\hat{EmpSE}}-1 \right)$ |
| Coverage in 95% CI | Proportion of simulations in which the confidence interval contains the true risk difference. | $\frac{1}{n_{sim}}\sum_{i=1}^{n_{sim}} 1(\hat{\theta}_{low,i}\leq\theta\leq\hat{\theta}_{upp,i})$ |

Abbreviations: CI, Confidence interval; EmpSE, Empirical SE; IQR, Interquartile range; RCT, Randomised controlled trial; SE, Standard error;

$\theta$ is the true risk difference; $\hat{\theta_{i}}$ is the estimated risk difference from simulation *i*; $\bar{\theta}$ is the mean of across repetitions*;*  $n_{sim}$ is the number of repetitions used; ModSE $\sqrt{\frac{1}{n_{sim}} \sum_{i=1}^{n_{sim}} \hat{Var}(\hat{\theta_{i})}}$;

**Reference**: Morris TP, White IR, Crowther MJ. Using simulation studies to evaluate statistical methods. Stat Med 2019; 38:2074–2102.

**Table S3. Proposed sample sizes for simulated trial designs where the number of patients in the control arm in the staggered platform trials with a concurrent control arm will continue running throughout the enrolment period for all 4 agents in the staggered platform trial.**

|  | **4 Standalone RCT’s** | **Parallel Platform Trial** | **Staggered Platform Trial (NCC)** | **Staggered Platform Trial (CCC)**  (AR = 1:1:1:1:1) | **Staggered Platform Trial (CCC)**  (AR = 2:1:1:1:1) | **Staggered Platform Trial (CCC)**  (AR = √*k*:1:1:1:1) |
| --- | --- | --- | --- | --- | --- | --- |
| **Scenario A (NIM 20%)** | | | | | | |
| Participants per treatment arm | 60 | 60 | 60 | 60 | 45 | 60 |
| Participants per control arm | 60 | 60 | 60 | >60 | >90 | >60 |
| **Total number of participants enrolled into the trials** | **480** | **300** | **300** | **>300** | **>270** | **>300** |
| **Scenario B (NIM 15%)** |  | | | | | |
| Participants per treatment arm | 93 | 93 | 93 | 93 | 71 | 93 |
| Participants per control arm | 93 | 93 | 93 | >93 | >141 | >93 |
| **Total number of participants enrolled into the trials** | **744** | **465** | **465** | **>465** | **>425** | **>465** |

Abbreviations: AR, Allocation Ratio; CCC, Concurrent Control; NCC, Nonconcurrent control; NIM, Non-inferiority margin; RCT, Randomised controlled trial.

**Figure S1. Stand-alone trial duration for Scenario A (20% NIM) and Scenario B (15% NIM).**

Abbreviations: IQR, Interquartile range;

Scenario A reflected a trial requiring 60 participants per arm, corresponding to a 20% non-inferiority margin. Scenario B reflected a trial requiring 93 participants per arm, corresponding to a 15% non-inferiority margin

**Table S4. Proportion and mean number of low control group comparisons in the staggered platform trial with a concurrent control arm under equal (1:1:1:1:1), unequal (2:1:1:1:1) and adaptive (√*k*:1:1:1:1) allocation ratios.** The target control arm size for each design is shown in the first column. For each drug-control comparison, the percentage of simulations in which the control arm was below the target and the mean control arm size across those simulations are presented.

| **Allocation ratio and**  **expected control arm size** | **Drug A vs Control** | | **Drug B vs Control** | | **Drug C vs Control** | | **Drug D vs Control** | |
| --- | --- | --- | --- | --- | --- | --- | --- | --- |
|  | **%** | **Mean** | **%** | **Mean** | **%** | **Mean** | **%** | **Mean** |
| **Scenario A** |  | | | | | | | |
| SPT - CCC 1:1:1:1:1  (Target control size 60) | 17·4 | 52·5 | 14·2 | 52·5 | 9·6 | 53·2 | 6·2 | 53·0 |
| SPT - CCC 2:1:1:1:1  (Target control size 90) | 17·9 | 78·9 | 15·5 | 78·8 | 12·1 | 80·0 | 10·1 | 80·2 |
| SPT - CCC √*k*:1:1:1:1 (Target control size 60) | 0·02 | 54·0 | 0·003 | 56·4 | 0·003 | 55·7 | 0·022 | 53·9 |
| **Scenario B** |  | | | | | | | |
| SPT - CCC 1:1:1:1:1  (Target control size 93) | 18·5 | 83·5 | 17·5 | 83·4 | 14·0 | 83·9 | 8·2 | 84·2 |
| SPT - CCC 2:1:1:1:1  (Target control size 141) | 17·7 | 127·2 | 17·1 | 127·2 | 14·8 | 127·7 | 10·9 | 128·3 |
| SPT - CCC √*k*:1:1:1:1  (Target control size 93) | 0·001 | 88·6 | 0·0001 | 85·5 | 0 | NA | 0·00025 | 87·8 |

Abbreviations: CCC, Concurrent control; SPT, Staggered platform trial;

**Table S5. Performance of the simulations for each treatment-control comparison and the overall trial design. Definitions for the performance measures can be found in Table S2.**

|  | Scenario A | | | | |  | | Scenario B | | | | | | |  |
| --- | --- | --- | --- | --- | --- | --- | --- | --- | --- | --- | --- | --- | --- | --- | --- |
| Trial design | Drug A | Drug B | Drug C | Drug D | Overall | |  | | Drug A | Drug B | Drug C | Drug D | | Overall | |
| Relative bias (%) (Monte Carlo standard error <1.8% for drug-control comparisons) | | | | | | | | | | | | | | |  |
| Stand-alone trials | 2.57 | 1·03 | -1.94 | -1.37 | 1·73 ^a^ | |  | | -1·20 | -1·51 | 0·46 | -0.26 | 0·86 ^a^ | | |
| Parallel platform trial | 2·52 | 1·67 | 4·42 | 2·02 | 2·66 ^a^ | |  | | -0·65 | -1·64 | -2·06 | -1·04 | 1·35 ^a^ | | |
| SPT - NCC | 0.58 | -1.82 | -0.94 | 0.84 | 1.04 ^a^ | |  | | -0·72 | -0.41 | -2·67 | -1·86 | 1·42 ^a^ | | |
| SPT – CCC (1:1:1:1:1) | -2·52 | -4·19 | -3·33 | -0·76 | 2·70 ^a^ | |  | | 0·68 | 2·17 | 0·36 | -0·80 | 1·00 ^a^ | | |
| SPT – CCC (2:1:1:1:1) | -0·50 | -0·84 | -0·34 | -0·64 | 0·58 ^a^ | |  | | 0·95 | -0·86 | 1·35 | 1·21 | 1·09 ^a^ | | |
| SPT – CCC (√*k*:1:1:1:1) | 0·38 | -3·91 | -1·10 | 1·22 | 1·65 ^a^ | |  | | -1·22 | 0·87 | -0·23 | 0·06 | 0·59 ^a^ | | |
| Empirical standard error | | | | | | | | | | | | | | |  |
| Stand-alone trials | 0·09 | 0·09 | 0·09 | 0·09 | 0·09 | |  | | 0·07 | 0·07 | 0·07 | 0·07 | 0·07 | | |
| Parallel platform trial | 0·09 | 0·09 | 0·09 | 0·09 | 0·09 | |  | | 0·07 | 0·07 | 0·07 | 0·07 | 0·07 | | |
| SPT - NCC | 0·09 | 0·09 | 0·09 | 0·09 | 0·09 | |  | | 0·07 | 0·07 | 0·07 | 0·07 | 0·07 | | |
| SPT – CCC (1:1:1:1:1) | 0·09 | 0·09 | 0·09 | 0·08 | 0·09 | |  | | 0·07 | 0·07 | 0·07 | 0·07 | 0·07 | | |
| SPT – CCC (2:1:1:1:1) | 0·09 | 0·09 | 0·09 | 0·09 | 0,09 | |  | | 0·07 | 0·07 | 0·07 | 0·07 | 0·07 | | |
| SPT – CCC (√*k*:1:1:1:1) | 0·08 | 0·08 | 0·08 | 0·08 | 0·08 | |  | | 0·06 | 0·06 | 0·06 | 0·06 | 0·06 | | |
| Relative % error in model-based standard error | | | | | | | | | | | | | | |  |
| Stand-alone trials | -0·89 | -0·79 | -1.49 | -0·06 | 0·81 ^a^ | |  | | -0·48 | -1·01 | 0·11 | 0.05 | 0·41 ^a^ | | |
| Parallel platform trial | -0·93 | -0·06 | -0·42 | -0·15 | 0·39 ^a^ | |  | | -1·04 | -0·36 | -0·45 | 0·34 | 0·54 ^a^ | | |
| SPT - NCC | -1·57 | -1.61 | -1·07 | -0.87 | 1.28 ^a^ | |  | | 0·13 | -0·73 | -0·57 | -0.24 | 0.28 ^a^ | | |
| SPT – CCC (1:1:1:1:1) | -0·18 | -0·83 | -1·04 | 0·55 | 0·65 ^a^ | |  | | -0·71 | -1·11 | -0·71 | -0·17 | 0·68 ^a^ | | |
| SPT – CCC (2:1:1:1:1) | -0·83 | -0·30 | -0·92 | -0·50 | 0·64 ^a^ | |  | | -0·68 | -0·25 | -0·80 | -0·42 | 0·54 ^a^ | | |
| SPT – CCC (√*k*:1:1:1:1) | -0·39 | -1·00 | -0·42 | -0·56 | 0·59 ^a^ | |  | | -0·84 | -0·26 | 0·17 | -0·09 | 0·34 ^a^ | | |
| Coverage in 95% CI (Monte Carlo standard error = 0.2%) | | | | | | | | | | | | | | |  |
| Stand-alone trials | 94.8 | 94·9 | 94·7 | 94.9 | 94·8 | |  | | 94·8 | 94·7 | 95·0 | 95·0 | 94·9 | | |
| Parallel platform trial | 94·7 | 94·9 | 95·0 | 94·8 | 94·8 | |  | | 94·7 | 95·1 | 94·7 | 95·2 | 94.9 | | |
| SPT - NCC | 94·7 | 94·3 | 94·8 | 94·6 | 94·6 | |  | | 95·1 | 94·6 | 94·8 | 95.0 | 94·9 | | |
| SPT – CCC (1:1:1:1:1) | 94·7 | 94·7 | 94·6 | 95·0 | 94·7 | |  | | 94·5 | 94·4 | 94·4 | 94·9 | 94·6 | | |
| SPT – CCC (2:1:1:1:1) | 94·6 | 94·4 | 94·4 | 94·4 | 94·5 | |  | | 94·8 | 95·0 | 94·8 | 94·8 | 94·8 | | |
| SPT – CCC (√*k*:1:1:1:1) | 94·8 | 94·8 | 94·8 | 94·5 | 94·7 | |  | | 94·7 | 95.0 | 94·8 | 94·9 | 94·9 | | |

Abbreviations: CCC, Concurrent control; FWER, Family wise error rate; NCC, Nonconcurrent control; SPT, Staggered platform trial; *Absolute mean

**Table S5 (Continued). Performance of the simulations for each treatment-control comparison and the overall trial design.**

|  | Scenario A | | | | |  | | Scenario B | | | | | | |  |
| --- | --- | --- | --- | --- | --- | --- | --- | --- | --- | --- | --- | --- | --- | --- | --- |
| Trial design | Drug A | Drug B | Drug C | Drug D | Overall | |  | | Drug A | Drug B | Drug C | Drug D | | Overall | |
| Mean squared error | | | | | | | | | | | | | | |  |
| Stand-alone trials | 0·008 | 0·008 | 0·008 | 0·008 | 0·008 | |  | | 0·005 | 0·005 | 0·005 | 0·005 | 0·005 | | |
| Parallel platform trial | 0·008 | 0·008 | 0·008 | 0·008 | 0·008 | |  | | 0·005 | 0·005 | 0·005 | 0·005 | 0·005 | | |
| SPT - NCC | 0·008 | 0·008 | 0·008 | 0·008 | 0·008 | |  | | 0·005 | 0·005 | 0·005 | 0·005 | 0·005 | | |
| SPT – CCC (1:1:1:1:1) | 0·008 | 0·008 | 0·007 | 0·007 | 0·007 | |  | | 0·005 | 0·005 | 0·005 | 0·005 | 0·005 | | |
| SPT – CCC (2:1:1:1:1) | 0·008 | 0·007 | 0·007 | 0·007 | 0·007 | |  | | 0·005 | 0·005 | 0·005 | 0·005 | 0·005 | | |
| SPT – CCC (√*k*:1:1:1:1) | 0·007 | 0·006 | 0·006 | 0·007 | 0·006 | |  | | 0·004 | 0·004 | 0·004 | 0·004 | 0·004 | | |

Abbreviations: CCC, Concurrent control; FWER, Family wise error rate; NCC, Nonconcurrent control; SPT, Staggered platform trial; *Absolute overall mean
